# Obesity affects the chondrocyte responsiveness to leptin in patients with osteoarthritis

**DOI:** 10.1186/ar3048

**Published:** 2010-06-09

**Authors:** Stéphane Pallu, Pierre-Jean Francin, Cécile Guillaume, Pascale Gegout-Pottie, Patrick Netter, Didier Mainard, Bernard Terlain, Nathalie Presle

**Affiliations:** 1 UMR S658 INSERM, Hôpital Porte Madeleine, 1 Rue Porte Madeleine, BP 2439, 45032 Orléans, France; 2 UMR CNRS-UHP 7561, Faculté de Médecine, Avenue de la forêt de Haye, BP 184, 54505 Vandoeuvre-les-Nancy, France

## Abstract

**Introduction:**

Increasing evidence support the regulatory role of leptin in osteoarthritis (OA). As high circulating concentrations of leptin disrupt the physiological function of the adipokine in obese individuals, the current study has been undertaken to determine whether the elevated levels of leptin found in the joint from obese OA patients also induce changes in the chondrocyte response to leptin.

**Methods:**

Chondrocytes isolated from OA patients with various body mass index (BMI) were treated with 20, 100 or 500 ng/ml of leptin. The expression of cartilage-specific components (aggrecan, type 2 collagen), as well as regulatory (IGF-1, TGFβ, MMP-13, TIMP 2) or inflammatory (COX-2, iNOS, IL-1) factors was investigated by real-time PCR to evaluate chondrocyte responsiveness to leptin. Furthermore, the effect of body mass index (BMI) on leptin signalling pathways was analyzed with an enzyme-linked immunosorbent assay for STATs activation.

**Results:**

Leptin at 20 ng/ml was unable to modulate gene expression in chondrocytes, except for MMP-13 in obese OA patients. Higher leptin levels induced the expression of IGF-1, type 2 collagen, TIMP-2 and MMP-13. However, the activity of the adipokine was shown to be critically dependent on both the concentration and the BMI of the patients with a negative association between the activation of regulated genes and BMI for 100 ng/ml of adipokine, but a positive association between chondrocyte responsiveness and BMI for the highest leptin dose. In addition, the gene encoding MMP-13 was identified as a target of leptin for chondrocytes originated from obese patients while mRNA level of TIMP-2 was increased in leptin-treated chondrocytes collected from normal or overweight patients. The adipokine at 500 ng/ml triggered signal transduction through a STAT-dependent pathway while 100 ng/ml of leptin failed to activate STAT 3 but induced STAT 1α phosphorylation in chondrocytes obtained from obese patients.

**Conclusions:**

The current study clearly showed that characteristics of OA patients and more expecially obesity may affect the responsiveness of cultured chondrocytes to leptin. In addition, the BMI-dependent effect of leptin for the expression of TIMP-2 and MMP-13 may explain why obesity is associated with an increased risk for OA.

## Introduction

Being obese is associated with elevated risks for an array of chronic diseases including osteoarthritis (OA). The effect of biomechanical loading on cartilage may explain part of the increased risk for knee OA in overweight people. However, the risk factor for the onset and progression of OA in non-weight-bearing joints such as hands has also been shown to be associated with body mass index (BMI) [1]. In fact, recent studies indicate that adiposity rather than simply excess body mass is detrimental to the knee joint and suggest that metabolic factors associated with adiposity may contribute to OA. The fat mass is associated with the loss of knee cartilage volume and the reduction in body fat is more closely related to symptomatic relief in knee OA than the loss of body weight [2-4]. In addition, Wang and colleagues demonstrated that the risk of primary knee and hip replacement for OA relates to both adipose mass and central adiposity [5].

Identifying the role of adipose-derived proteins in the development and progression of joint disorders has been the aim of increasing investigations, and recent studies propose leptin as a key mediator linking obesity to OA [2,6,7]. Among adipokines found in the synovial fluid obtained from OA patients, leptin is the only one for which joint levels exceed those determined in paired serum [8,9]. The expression of leptin and its functional receptor is strongly up-regulated in human OA cartilage and is related to the grade of cartilage destruction [9,10]. Although leptin may be a local factor able to modulate chondrocyte functions, its contribution in OA remains unclear. The adipokine stimulates the expression of growth factors and the synthesis of extracellular matrix [10,11]. Beside this stimulatory effect on cartilage production, leptin modulates the degradative functions of the chondrocytes through up-regulation of matrix metalloproteases (MMP)-9 and -13 and interleukin (IL)-1 [9,12]. Moreover, leptin was shown to enhance the synthesis of proinflammatory mediators in human OA cartilage [13] and to potentiate the IL-1-mediated production of nitric oxide, which is known to contribute to cartilage matrix loss [14]. Whatever these ambivalent effects, the most important evidence for the role of leptin in the development of OA is the lack of any increased spontaneous degenerative changes in obese mice with impaired leptin signalling compared with lean wild-type mice, indicating that leptin is required to increase the incidence of OA due to extreme adiposity [7].

Although previous studies have addressed the potential role of leptin in OA, the influence of BMI on chondrocyte response to the adipokine has not been investigated. Obese OA patients exhibit elevated leptin levels in synovial fluid and a high expression of leptin in cartilage compared with lean patients, indicating that local hyperleptinemia may be found in the joint. Interestingly, obesity is characterized by a systemic hyperleptinemia whereas leptin should promote weight loss through its effects on food intake and energy expenditure in the hypothalamus. This defect in leptin action in obese individuals suggests that human obesity may be associated with central resistance to leptin [15]. The present study was therefore designed to determine whether such loss of leptin activity also occurs in cartilage. For this purpose, chondrocytes isolated from OA patients with various BMIs ranging from 22 to 40 kg/m^2 ^were treated with various concentrations of leptin (20, 100 and 500 ng/ml). RT-PCR analysis for genes encoding cartilage components (aggrecan and type 2 collagen) as well as factors involved in matrix remodeling, that is insulin growth factor-1 (IGF-1), transforming growth factor-β (TGFβ), MMP-13 and tissue inhibitors of metalloproteinase (TIMPs), or in inflammation, that is cyclooxygenase-2 (COX-2), inducible nitric oxide synthase (iNOS) and IL-1, were then performed to evaluate chondrocyte responsiveness to leptin.

## Materials and methods

### Isolation and treatment of human chondrocytes

Articular cartilage samples were obtained from femoral condyles and tibial plateaus of patients undergoing total knee replacement surgery (n = 25; mean age 68.9 ± 9.5 years, range 52 to 82 years; mean BMI 31 ± 5.3 kg/m^2^, range 22.5 to 40.3 kg/m^2^). Knee OA was diagnosed from clinical and radiologic evaluations based on the American College of Rheumatology criteria [16]. Detailed clinical data were obtained from patients regarding grade of physical activity, BMI, presence of pain, and stiffness and swelling of the joint. The study protocol conformed to the ethical guidelines of the Declaration of Helsinki, and written informed consent was obtained from each participant.

Chondrocytes were isolated after a sequential digestion of cartilage tissues with pronase (0.15%, w/v) (Roche Applied Science, Mannheim, Germany) for three hours at 37°C and collagenase B (0.2%, w/v) (Roche Applied Science, Mannheim, Germany) overnight at 37°C. After centrifugation of the resulting cells and suspension in Dulbecco's Modified Eagles Medium/Ham's F-12 (Invitrogen, Cergy-Pontoise, France) supplemented with 5% (v/v) fetal calf serum, 100 U/ml penicillin-streptomycin, and 2 mM L-glutamine (Invitrogen, Cergy-Pontoise, France), chondrocytes were expanded at 37°C in a monolayer in a humidified atmosphere containing 5% carbon dioxide until reaching confluence. Human chondrocytes were seeded thereafter in six-well plates. After overnight starvation in serum-free medium and subsequent change of serum-free culture medium two hours before leptin treatment, chondrocytes were incubated in serum-deprived medium with or without human recombinant leptin (20, 100 or 500 ng/ml) (R&D Systems, Abingdon, UK) for 24 hours.

### RNA isolation and real-time reverse transcriptase-polymerase chain reaction

Total RNA was extracted from chondrocytes using the RNeasy Mini Kit (Qiagen, Courtaboeuf, France). The integrity of the isolated RNA was assessed by ethidium bromide staining on agarose gel and the concentration was determined by measurement of the optical density at 260 nm on a NanoDrop ND-1000 Spectrophotometer (Labtech, Palaiseau, France). RNA samples were then reverse-transcribed for 1.5 hours at 37°C to complementary DNA using Moloney murine leukemia virus reverse transcriptase (M-MLV-RT; 200 U; Gibco BRL, Cergy-Pontoise, France), deoxynucleotide triphosphates (dNTP; 5 mM), DTT (0.1 M) and anchored 15 mer oligo-dT primers (100 pmol; MWG biotech SA, Courtaboeuf, France).

Gene expression was analyzed by quantitative real-time PCR (Lightcycler, Roche, Mannheim, Germany) using the SYBRgreen master mix system (Qiagen, Courtaboeuf, France). The conditions for amplification were: initial activation step at 95°C for 15 minutes, denaturation at 94°C for 45 seconds, hybridization of primers at defined temperature Tm for 45 seconds and elongation at 72°C for 1 minute. The gene-specific primer pairs optimized for this method, the Tm as annealing temperature and the corresponding product size are summarized in Table 1. As a control of the amplification specificity, melting curve analysis was performed for each PCR experiment to separate the specific product from the non-specific products (if any). Each run included positive and negative controls. For standardization of gene expression levels, mRNA ratios relative to RP29 as housekeeping gene were calculated, and real-time quantitative data were analyzed using the ΔΔCt method.

**Table 1:** Thermal cycling conditions for real time PCR

Gene	Sequences	Temp (°C)	Amplicon size (bp)
Aggrecan	Fw TCG AGG ACA GCG AGG CC Rv TCG AGG GTG TAG CGT GTA GAG A	61	85
			
Collagen type 2	Fw ATG ACA ATC TGG CTG CCA Rv CTT CAG GGC AGT GTA CGT	58	200
			
IGF-1	Fw GTA TTG CGC ACC CCT CAA Rv TTG TTT CCT GCA CTC CCT CT	57	126
			
TGFβ	Fw TGC GGC AGC TGT ACA TTG A Rv TGG TTG TAC AGG GCC AGG A	59	186
			
MMP-13	Fw TGG TGG TGA TGA AGA TGA TTT G Rv TCT AAG CCG AAG AAA GAC TGC	57	125
			
TIMP-2	Fw CTT CTT TCC TCC AAC GTC Rv AAA GCG GTC AGT GAG AAG GA	57	183
			
COX-2	Fw GCT GGA ACA TGG AAT TAC CCA Rv CTT TCT GTA CTG CGG GTG GAA	58	98
			
iNOS	Fw ACA AGC CTA CCC CTC CAG AT Rv TCC CGT CAG TTG GTA GGT TC	59	157
			
IL-1	Fw GGA CAA GCT GAG GAA GAT GC Rv TCG TTA TCC CAT GTG TCG AA	57	120
			
RP 29	Fw AAG ATG GGT CAC CAG CAG GTC TAC TG Rv AGA CGC GGC AAG AGC GAG AA	59	153

bp, base pairs; COX-2, cyclooxygenase-2; IGF-1, insulin growth factor-1; MMP, matrix metalloproteinase; iNOS, inducible nitric oxide synthase; Temp, temperature; TGF, transforming growth factor; TIMP, tissue inhibitor of metalloproteinase; IL-1, interleukin-1.

### Determination of STATs activation

After treatment of chondrocytes with leptin (100 or 500 ng/ml) for 10 minutes, the nuclear extracts were prepared using the Nuclear Extract kit (Active motif, Rixensart, Belgium). Protein concentration was measured by the BCA protein assay. The activation of the signal transducer and activator of transcription (STAT) pathway by leptin was then monitored using an ELISA-based kit specific for STAT 1α, STAT 3, STAT 5A et 5B (TransAM STAT family kit, Active motif, Rixensart, Belgium).

### Data analysis

Results were expressed as means ± standard error of the mean of at least three measurements. Statistical analysis was conducted with SPSS software (SPSS Inc., Chicago, IL, USA). Comparisons between treated and untreated chondrocytes were analyzed using the non-parametric U-Mann Whitney test. The Spearman's rho correlation method was used to evaluate the relation between gene expression and the BMI. A *P *value less than 0.05 was considered significant for differences and correlations.

## Results

The effect of various leptin treatments was determined in human OA chondrocytes categorized in two groups according to the BMI value of the patients from whom they originated. The treatment group details showing information on sample size, sex distribution, age and BMI are reported in Table 2.

**Table 2:** Details of sample size, sex distribution, age and BMI for each leptin treatment group

	Leptin 20 ng/ml		Leptin 100 ng/ml		Leptin 500 ng/ml	
			
	BMI <30	BMI >30	BMI <30	BMI >30	BMI <30	BMI >30
N	5 (3 F, 2 M)	5 (2 F, 3 M)	8 (5 F, 3 M)	11 (7 F, 4 M)	8 (5 F, 3 M)	9 (6 F, 3 M)
Age (years)	69.4 (4.5)	67.8 (4.5)	71.3 (4.1)	68 (2.4)	68.4 (3.5)	67.7 (2.6)
BMI (kg/m^2^)	26.04 (1.02)	33.38 (1.85)	26.78 (0.78)	34.45 (1.10)	26.24 (0.68)	35.44 (1.31)

Values are expressed as mean (standard error of the mean). BMI, body mass index; F, female; M, male.

### Baseline gene expression in non-stimulated chondrocytes

**Figure 1:**
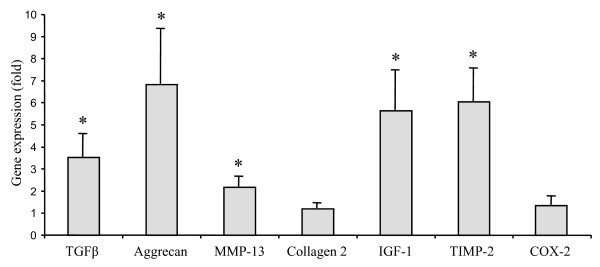
**Effect of BMI on the constitutive expression of TGFβ, aggrecan, MMP-13, type 2 collagen, IGF-1, TIMP-2 and COX-2 in chondrocytes obtained from OA patients**. mRNA levels were determined 24 hours after culture medium change by quantitative real-time PCR. For every patient, experiments was performed in triplicate. Results were expressed as mean ± standard error of the mean of variation found in obese osteoarthritis (OA) patients (body mass index (BMI) >30 kg/m^2^, n = 13) over values obtained in normal or overweight OA patients (BMI <30 kg/m^2^, n = 12). * *P *< 0.05 between obese and normal or overweight patients. COX-2, cyclooxygenase-2; IGF-1, insulin growth factor-1; MMP, matrix metalloproteinase; TGFβ, transforming growth factor-β; TIMP, tissue inhibitor of metalloproteinase.

Before examining leptin effects, we investigated the baseline gene expression pattern in both groups. The results indicated that non-stimulated chondrocytes obtained from obese patients overexpressed IGF-1, TGFβ, aggrecan and TIMP-2 compared with cells provided by normal or overweight patients (Figure 1). The expression of MMP-13 was slightly but significantly increased in patients with BMI of more than 30 kg/m^2^. By contrast, no significant difference was observed for type 2 collagen or COX-2, and we failed to detect mRNA for iNOS and IL-1 in untreated cells.

### Effect of leptin on gene expression in OA chondrocyte

The recombinant adipokine induced a change in the expression of genes encoding cartilage-specific elements and regulating factors depending on the concentration applied and the BMI of the patients.

**Figure 2:**
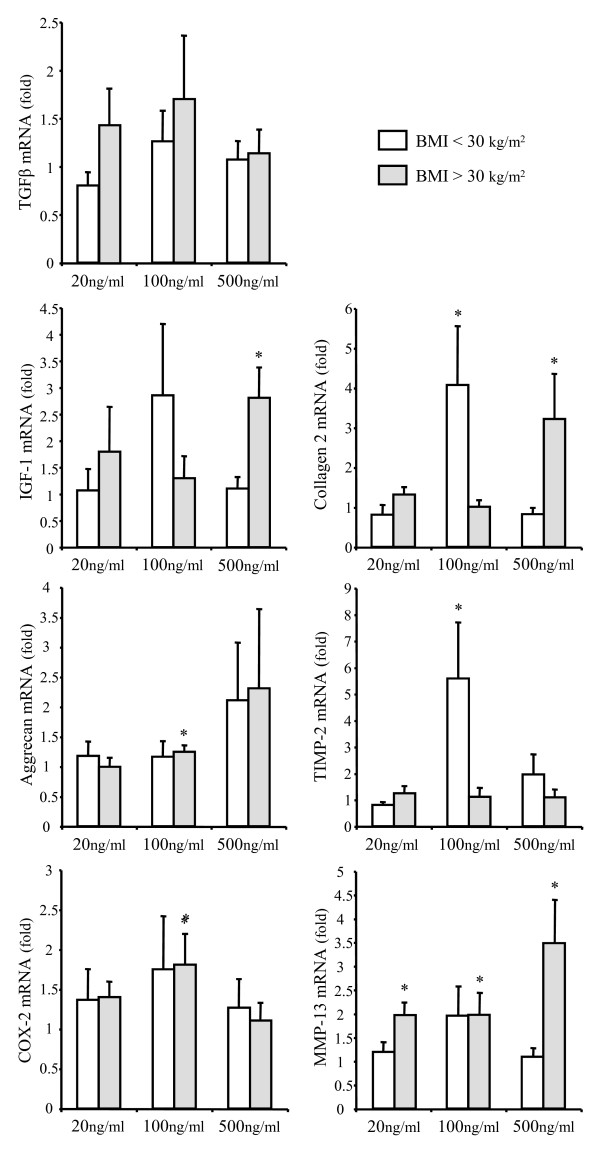
**Effect of leptin on the expression of TGFβ, IGF-1, type 2 collagen, aggrecan, TIMP-2, COX-2 and MMP-13 in chondrocytes obtained from normal or overweight and obese OA patients**. Normal or overweight osteoarthritis (OA) patients: body mass index (BMI) <30 kg/m^2^; n = 5 for 20 ng/ml and n = 8 for 100 and 500 ng/ml. Obese OA patients: BMI >30 kg/m^2^; n = 5 for 20 ng/ml, n = 11 for 100 ng/ml and n = 9 for 500 ng/ml. Quantitative real-time PCR analysis were performed 24 hours after exposure of chondrocytes to 20, 100 or 500 ng/ml of leptin. For every patient, experiments were carried out in triplicate and results were expressed as means ± standard error of the mean over control values. * *P *< 0.05 between leptin-treated and control chondrocytes. COX-2, cyclooxygenase-2; IGF-1, insulin growth factor-1; MMP, matrix metalloproteinase; TGFβ, transforming growth factor-β; TIMP, tissue inhibitor of metalloproteinase.

Leptin at 20 ng/ml was unable to significantly modulate gene expression in chondrocytes, except for MMP-13 in obese OA patients (Figure 2).

The RT-PCR analysis showed a dose-dependent effect of leptin on the expression of type 2 collagen. mRNA was strongly overexpressed in chondrocytes collected from normal or overweight patients upon treatment with 100 ng/ml of leptin. However, this stimulatory effect did not exist at 500 ng/ml. By contrast, the highest concentration of leptin was required to up-regulate type 2 collagen in chondrocytes isolated from obese patients (Figure 2).

Treatment with 100 ng/ml of leptin of chondrocytes originated from patients with BMI of more than 30 kg/m^2 ^slightly but significantly increased the expression of aggrecan (Figure 2). An elevated level of aggrecan mRNA was also found at 500 ng/ml of leptin for both BMI patient groups, but the difference with unstimulated cells did not reach statistical significance.

The effect of leptin on the expression of growth factors was different between IGF-1 and TGFβ. The expression of TGFβ remained unchanged in both groups of patients whatever the leptin concentration used. By contrast, leptin induced the expression of IGF-1 with a pattern of response similar to that found for type 2 collagen. The adipokine at 100 ng/ml increased the mRNA level of IGF-1 in chondrocytes obtained from normal or overweight patients (Figure 2). However, this stimulatory effect was not shown at higher concentration. Chondrocytes from obese OA patients exhibited an opposite response to the adipokine with a lack of significant effect on IGF-1 expression at 100 ng/ml but a strong up-regulation of IGF-1 at 500 ng/ml of leptin (Figure 2).

The gene encoding MMP-13 was not identified as a significant target of leptin for chondrocytes originated from normal or overweight patients. By contrast, leptin dose-dependently induced the expression of the degradative enzyme in chondrocytes obtained from obese patients (Figure 2).

The expression of TIMP-2 in chondrocytes from normal or overweight patients was highly up-regulated upon treatment with 100 ng/ml of leptin, as a more than five-fold increase over the control value was achieved with this concentration of adipokine. However, the adipokine did not show any effect when cells derived from patients with BMI of more than 30 kg/m^2 ^were stimulated with 500 ng/ml of leptin. Similarly, leptin did not induce any change in the mRNA level of TIMP-2 in cells isolated from obese patients (Figure 2).

Further investigations on the effect of leptin on the expression of inflammatory mediators indicated that the adipokine was not able to modulate the mRNA level of iNOS and IL-1. COX-2 was slightly up-regulated by leptin, but the difference with untreated cells reached statistical significance only with chondrocytes obtained from obese patients and stimulated with 100 ng/ml of leptin.

### Role of BMI on leptin effects

**Figure 3:**
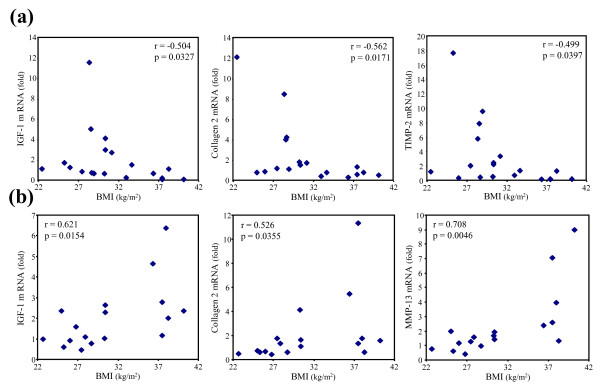
**Relations between chondrocyte responsiveness to leptin and BMI of OA patients**. Chondrocytes were exposed to leptin at **(a) **100 ng/ml or **(b) **500 ng/ml and mRNA levels for type 2 collagen, insulin growth factor-1 (IGF-1), tissue inhibitor of metalloproteinase (TIMP)-2 and matrix metalloproteinase (MMP)-13 were determined by quantitative real-time PCR. For every patient, experiment was carried out in triplicate, and results were expressed as means over control values. Correlations were calculated by Spearman's correlation analysis (r, correlation coefficient with p value of correlation). *P *< 0.05 was considered significant.

The effect of leptin on gene expression was shown to be dependent on the BMI of patients from whom the cells were originating. The gene encoding TIMP-2 was induced in chondrocytes obtained from normal or overweight patients while mRNA level of MMP-13 or COX-2 was increased in leptin-treated chondrocytes collected from obese OA patients. In addition, the BMI changed the leptin dose-response of chondrocytes. The responsiveness found with 100 ng/ml of leptin in chondrocytes obtained from normal or overweight patients was not present at the highest concentration of adipokine. By contrast, leptin up-regulated gene expression in a dose-dependent manner in chondrocytes collected from obese OA patients as cells required an elevated level of leptin to be responsive (Figure 2). Based on this BMI-dependent leptin dose-response of chondrocytes, we demonstrated that the stimulatory effect of leptin at 100 ng/ml on the expression of IGF-1, collagen type 2 and TIMP-2 was negatively related to the BMI of the patients while a positive association between chondrocyte responsiveness and BMI was found at 500 ng/ml of leptin for IGF-1, collagen type 2 and MMP-13 (Figure 3).

### Effect of leptin on the STAT pathway

**Figure 4:**
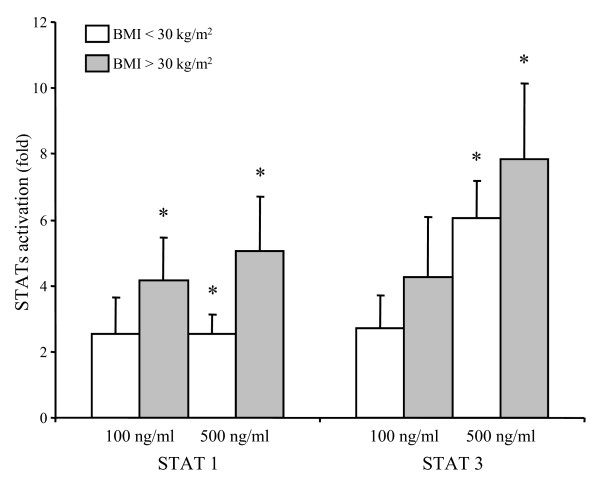
**Effect of leptin on the activation of the STAT pathway in chondrocytes obtained from normal or overweight (BMI <30 kg/m^2^) and obese (BMI >30 kg/m^2^) OA patients**. The phosphorylation of STAT 1α, -3, -5A and -5B was determined with nuclear proteins obtained from chondrocytes exposed to leptin at 100 ng/ml or 500 ng/ml for 10 minutes. For every patient, experiments were carried out in duplicate and results were expressed as means ± standard error of the mean over control values. * *P *< 0.05 between leptin-treated and control chondrocytes. BMI, body mass index; OA, osteoarthritis; STAT, signal transducer and activator of transcription.

The activation of STAT has been investigated to determine whether BMI may affect the leptin signalling pathway. The highest adipokine concentration induced an early activation of STAT 1α and STAT 3 (Figure 4). By contrast, the phosphorylation of STAT 5A and 5B in leptin-stimulated chondrocytes remained unchanged compared with untreated cells (data not shown). Leptin at 100 ng/ml was unable to activate the STAT pathway, except for STAT 1α in chondrocytes collected from OA patients with BMI of more than 30 kg/m^2^. The level of STAT activation increased with the BMI, but the difference between both groups of patients did not reach statistical significance.

## Discussion

As a defect in leptin action on body weight homeostasis has been reported for obese individuals despite high serum leptin concentrations, the current study was undertaken to determine whether chondrocytes from obese OA patients that are exposed to elevated leptin levels, exhibit an altered response to leptin.

Before examining the effect of BMI on chondrocyte responsiveness to leptin, we compared the gene expression pattern between obese and non obese OA patients. The RT-PCR analysis showed major changes between both groups with an elevated mRNA level of growth factors, aggrecan and TIMP-2 in chondrocytes from obese patients suggesting that the chondrocyte metabolic activity is increased with obesity. Such overexpression of TGFβ, IGF-1 and TIMP-2 has been previously reported in subcutaneous adipose tissue from obese individuals [17,18] indicating that chondrocytes may also exhibit an obesity-related gene expression pattern. These findings suggest also that a pathological status may result in a similar pattern of expression of some factors in both chondrocytes and adipocytes. As obesity and OA are both associated with inflammation, the up-regulation of growth factors and MMP inhibitors may be an attempt at an adaptive response to an inflammatory environment. However, we failed to detect any difference in the expression of proinflammatory mediators between non-obese and obese OA patients, suggesting that the chronic low grade of inflammation associated with obesity rather than the OA-related joint inflammation is involved in the overexpression of TGFβ, IGF-1 and TIMP-2 in both chondrocytes and adipocytes.

The downstream targets of leptin identified in the current study are in agreement with those previously published because IGF-1, type 2 collagen and MMP-13 were overexpressed upon leptin stimulation [9-12]. However, conflicting results were found for the effect of leptin on the expression of inflammatory mediators. Although Vuolteenaho and colleagues showed that leptin enhanced the expression of iNOS in human OA cartilage [13], iNOS mRNA level remained unchanged in leptin-treated cells. The use of cartilage explants instead of isolated chondrocytes may explain the discrepancies because other authors were unable to find any effect of leptin on the expression of iNOS in cultured human primary chondrocytes [9,14]. Similarly, we failed to detect any change in the expression of IL-1 whereas Simopoulou and colleagues reported a stimulatory effect of leptin on the production of the cytokine [9]. In fact, human chondrocytes required long-term cultures to produce IL-1 suggesting an indirect leptin-mediated pathway.

One of the most relevant findings arising from this study is the BMI-dependent effect of leptin on the expression of the genes encoding TIMP-2 and MMP-13 in chondrocytes. Our results provided new insights on the role of leptin in matrix remodeling by regulating the balance between MMPs and TIMPs, which are the physiologic protein inhibitors of the degradative enzymes. Although TIMP-1 is highly expressed in cartilage and reduced in OA, we failed to detect any effect of leptin on its expression (data not shown). By contrast, mRNA levels for TIMP-2 was markedly increased in leptin-treated chondrocytes compared with control cells. However, this up-regulation of TIMP-2 was found in normal or overweight patients only, and decreased when the BMI of the patients increased. Besides, MMP-13 was overexpressed in leptin-stimulated chondrocytes obtained from obese OA patients, and this was shown from the lowest concentration of leptin. These BMI-dependent effects of leptin may change the degenerative process during OA. The leptin-induced TIMP-2 expression may delay cartilage destruction in non-obese OA patients while the adipokine may enhance cartilage damage in obese OA patients. It is worth noting that the BMI-dependent effect of leptin found for genes involved in matrix remodeling was not showed for COX-2, iNOS or IL-1 suggesting that the adipokine did not modulate the production of proinflammatory mediators in cartilage from obese patients.

The other interesting finding of the study is the influence of leptin concentration. Among the three concentrations used in the current study, two doses represented the *in vivo *situation given that the synovial fluid levels of leptin in patients with OA range from 1 to 100 ng/ml [8]. As the chondrocyte sensitivity to exogenous stimulating factor may be reduced when cells are isolated from the extracellular matrix, a higher leptin dosage was also tested [19]. Most of the anabolic and catabolic genes were insensitive to the lowest dose of leptin regardless of BMI, MMP-13 being the single target of leptin at 20 ng/ml in obese patients. Beside, the relations between cell responsiveness and BMI indicated that chondrocytes collected from patients with low BMI displayed most responsiveness with 100 ng/ml of leptin. This dosage also induced the expression of genes encoding aggrecan, COX-2 and MMP-13 in obese patients. However, we were not able to find any significant correlation between cell responsiveness and BMI with these slight stimulatory effects of leptin at 100 ng/ml. The lack of a marked effect of leptin at 20 ng/ml also explains the lack of any association between chondrocyte response to the adipokine and BMI. The most striking effects of leptin in obese patients, that is the up-regulation of IGF-1, collagen type 2 and MMP-13, were found with the highest concentration of leptin. Chondrocytes from obese patients may already be so strongly exposed to elevated leptin levels within the joint that they are refractory to further stimulation. The attenuation of leptin sensitivity may result either from a downregulation of the leptin receptor or from impairments of the signal transduction process. In agreement with the data obtained by Simopoulou and colleagues [9], we failed to detect any change in the expression of the receptor neither between normal or overweight and obese patients, nor upon stimulation of OA chondrocytes with 100 or 500 ng/ml of leptin (data not shown). The activation of the Janus kinase/STAT pathway has been investigated to determine whether the leptin-induced signal transduction process was not impaired [20]. In the current study, the elevated concentration of leptin actually induced the phosphorylation of STAT 1α and -3 in chondrocytes. This STAT activation resulted in the expression of target genes in chondrocytes issued from obese patients but was unable to drive gene expression in cells collected from normal or overweight patients probably because of a negative feedback inhibition of leptin signaling. By contrast, the adipokine at 100 ng/ml mediated its effects through a STAT-independent pathway because this concentration of leptin failed to activate STAT 3. Other signaling pathways, such as ERK and phosphatidylinositol3-kinase, may be involved. Leptin was shown to regulate the differentiation of the ATDC5 chondrogenic cell line through activation of ERK1/2 [21], and to activate the IRS-1/PI3K/Akt pathway to induce migration of human chondrosarcoma cells [22]. The STAT-independent signal transduction process was, however, disrupted when chondrocytes from obese OA patients were stimulated with 100 ng/ml of leptin. As STAT 1 has been found to act as a negative transcriptional regulator [23,24], leptin-induced STAT 1 phosphorylation in chondrocytes from obese patients may interfere with the transcriptional machinery and may lead to the loss of cell sensitivity to leptin. The responsiveness to the adipokine was then restored in chondrocytes from obese patients after activation of STAT 3 by high adipokine level. The relations found in the current study between BMI and the leptin-induced gene expression, which are negative for 100 ng/ml of leptin and positive for the highest leptin level, further support the finding that leptin may have the ability to differentially regulate the expression of target genes depending on both the BMI and the dose.

## Conclusions

The current study indicated that MMP-13 was the single target gene induced by the 20 ng/ml leptin treatment. More importantly, chondrocytes from obese OA patients exhibit a response pattern to leptin different from those collected from normal or overweight patients. Consequently, leptin may protect cartilage from the degenerative process in patients with BMI of more than 30 kg/m^2 ^through overexpression of genes encoding IGF-1, type 2 collagen and TIMP-2. By contrast, leptin may have a dualistic effect in obese patients as it up-regulated not only IGF-1 and type 2 collagen but also MMP-13. This BMI-dependent effect of leptin may explain why obesity is a risk factor for OA and further supports the recent data found by Griffin and colleagues, which showed that the incidence of knee OA is not increased in extremely obese leptin-impaired signaling [7]. The current findings provide therefore new insights to better understand the relation between obesity and OA, and to use a preventive approach with dietetic guidelines to reduce the incidence of OA. They are also a new starting point to identify the molecular mechanism for the BMI-dependent regulation of gene expression by leptin.

## Abbreviations

BMI: body mass index; COX-2: cyclooxygenase-2; ELISA: enzyme-linked immunosorbent assay; IGF-1: insulin growth factor-1; IL-1: interleukin-1; MMP: matrix metalloproteinase; iNOS: inducible nitric oxide synthase; OA: osteoarthritis; RT-PCR: reverse-transcription polymerase chain reaction; STAT: signal transducer and activator of transcription; TGFβ: transforming growth factor-β; TIMP: tissue inhibitor of metalloproteinase.

## Competing interests

The authors declare that they have no competing interests.

## Authors' contributions

SP carried out the experiments and the RT-PCR analysis, performed the statistical analysis, participated in the interpretation of the data and drafted the manuscript. PJF was involved in collecting and preparing samples, performed the experiments and participated in the interpretation of the data. CG was involved in collecting and preparing samples, and carried out RT-PCR experiments. PP participated in the design of the study and helped to draft the manuscript. PN helped to draft the manuscript. DM recruited the patients and provided the cartilage specimens and helped to draft the manuscript. BT was involved in interpretating the data and revising the manuscript critically for important intellectual content. NP participated in the coordination of the study and in the interpretation of the data, and drafted the manuscript. All authors read and approved the final version to be published.
